# A Case of Anaemia With High-Grade Splenomegaly

**DOI:** 10.7759/cureus.24908

**Published:** 2022-05-11

**Authors:** Ranjan K Singh

**Affiliations:** 1 Internal Medicine, Antiretroviral Therapy Centre, District Hospital, Khagaria, IND

**Keywords:** antioxidants, thalassaemia, splenomegaly, haemoglobin e, severe anaemia

## Abstract

In tropical areas, there are a variety of parasitic and nonparasitic causes of high-grade splenomegaly. An adolescent male patient with haemoglobin E/β-thalassaemia came with high-grade splenomegaly and severe anaemia, requiring blood transfusions on a regular basis. Treatment with folic acid and antioxidant vitamins reduced the requirement for blood transfusions, brought haemoglobin levels back to near normal, and reduced splenic enlargement.

Haemoglobin E/β-thalassaemia is a haematological condition that causes anaemia and high-grade splenomegaly in the tropics. Initially, the disease was only seen in Southeast Asia, but it has since spread around the world due to migration from that region.

## Introduction

Splenomegaly is classified into five grades depending on the enlargement below the left costal margin, as follows: grade 0, spleen not palpable; grade 1, spleen palpable inside left costal margin during inspiration; grade 2, spleen palpable below left costal margin; grade 3, spleen enlarged halfway between left costal margin and umbilicus; grade 4, spleen enlarged to the umbilicus; grade 5, spleen enlarged to iliac crest [1].

Although parasitic infections, such as chronic malaria, schistosomiasis, and visceral leishmaniasis, are the major causes of massive splenomegaly in tropical regions, haematological disorders such as thalassaemia, particularly haemoglobin E/β-thalassaemia, are a frequent cause of splenomegaly in Southeast Asia, Bangladesh, and India's north-eastern area. In north India, however, there are few examples. On the other hand, individual migration has spread the disease globally [2,3]. Haemoglobin E (Hb E) is abnormal haemoglobin caused by a point mutation in the β-globin gene base CAG ® AAG at codon 26 that replaced glutamic acid for lysine [4,5]. The homozygous state of Hb EE is asymptomatic, whereas the heterozygous state, that is, Hb E/-thalassaemia mimics β-thalassaemia intermedia. Its severity ranges from mild to severe based on independent parameters such as age at presentation, steady haemoglobin, splenic enlargement, age at first blood transfusion, blood transfusion requirement, and growth/development [6].

## Case presentation

**Figure 1:**
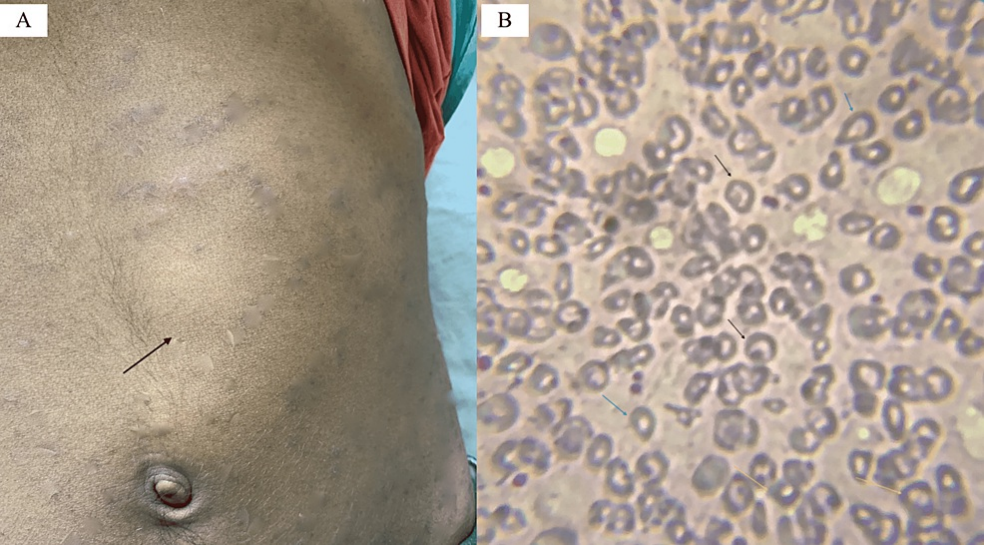
(A) The anterior notch of the spleen; (B) Peripheral blood smear stained with Leishman under 200x magnification shows target cells, teardrop-shaped cells, and basophilic stippling of RBCs with arrows in black, blue, and yellow, respectively.

A young man in his early twenties presented with severe anaemia and a huge lump on the left side of his abdomen. He was afebrile and did not show any sign of growth retardation. His parents were both in good health. He has, however, received multiple blood transfusions in the past 3-4 years. The patient had severe pallor, mild icterus, an enlarged liver (3 cm below the right costal margin), and an enlarged spleen towards the umbilicus (10 cm below the left costal margin) on clinical examination (Figure 1A). In addition, microcytic hypochromic RBCs with multiple target cells, teardrop-shaped cells, and RBCs with basophil stippling were seen in a peripheral blood smear (Figure 1B).

**Figure 2:**
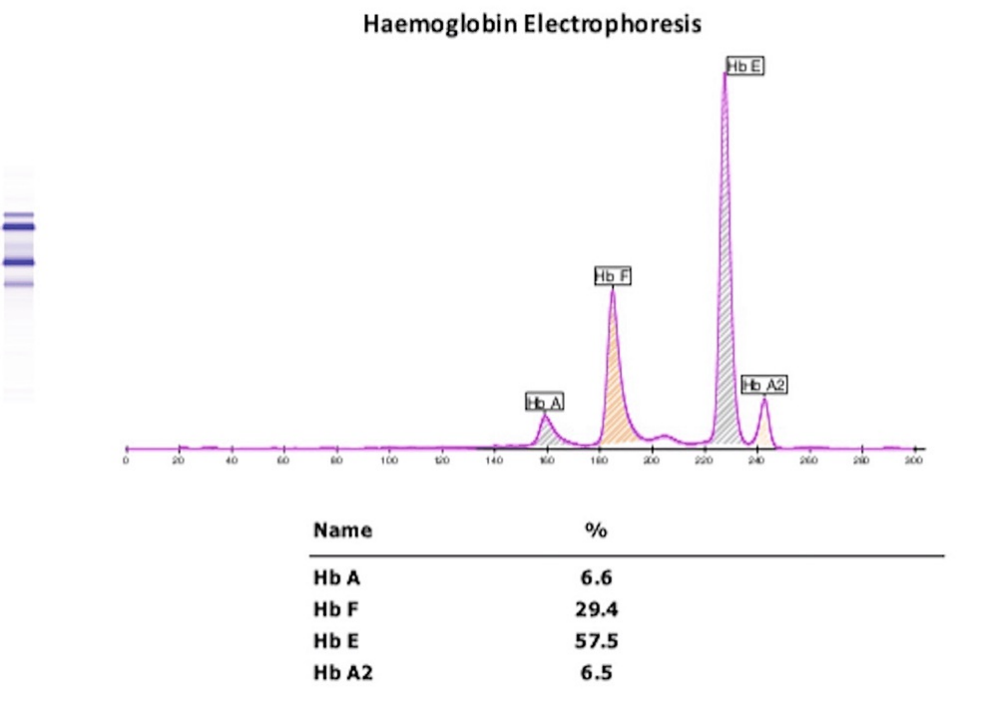
Capillary zone electrophoresis of haemoglobin.

Rapid antigen test for Plasmodium vivax and Plasmodium falciparum malaria and antibodies for Leishmania donovani in visceral leishmaniasis using an immunochromatographic RK-39 strip were negative. The patient’s mean corpuscular volume was 66 fL (ref. 82-90); serum ferritin and serum iron levels were 1200 ng/mL (ref. 236) and 76.7 mg/dL (ref. 60-160), respectively. Additionally, Hb A, Hb A2, Hb F, and Hb E levels were found to be 6.6%, 6.5%, 29.4%, and 57.5%, respectively, by capillary zone electrophoresis (CZE), indicative of a combination of β-thalassaemia and Hb E haemoglobinopathy (Figure 2).

The patient was given 1 mg of folic acid per day and antioxidants, including vitamin E (400 units) and vitamin C (500 mg), on a regular basis. After six months, haemoglobin increased to 9.0 g/dL, and serum ferritin was 321 ng/dL. The size of the spleen shrank to 6 cm below the left costal edge. The patient did not require additional blood transfusions.

## Discussion

Microcytic anaemia is seen in iron deficiency, thalassaemia, lead poisoning, sideroblastic anaemia, and anaemia relating to chronic diseases. An MCV of <75 fL and raised ferritin are seen in thalassaemia but not in other causes of microcytic anaemia. Target cells are seen in thalassaemia, anaemia associated with liver disease, and after splenectomy. In contrast, teardrop-like cells are seen in anaemia relating to renal diseases, cancers, myelofibrosis, and β-thalassaemia. Although basophil stippling in the peripheral smear is associated with lead poisoning, it can also be found in haemoglobinopathies and myelodysplasia. The electropherogram of healthy adults shows Hb A levels of 96.8-97.8%, Hb F levels of 0.0-0.5%, Hb A2 levels of 2.2-3.2%, and Hb E levels of 0%. In contrast, β-thalassaemia is characterized by decreased or absent Hb A and elevated levels of HB A2 and Hb F [3]. Hb E/β-thalassaemia intermedia is microcytic anaemia with milder symptoms that appear later in life [7]. High Hb F levels indicate a survival advantage in Hb E disease [8]. This case has grade 4 splenomegaly according to Hackett's grading of the palpable spleen and the severity of the disease. Based on six independent parameters, it is within a moderate range [1,6]. Splenomegaly is a common complication of symptomatic thalassaemia, and it exacerbates anaemia. Other factors contributing to the aetiology of anaemia include inefficient erythropoiesis, apoptosis, oxidative damage, and limited red cell survival. 

Hb E has significantly decreased oxygen affinity compared to other types of β-haemoglobinopathy [8], which is why a fixed level of haemoglobin in Hb E disease is of limited value for determining the need for blood transfusion [9]. A recent cohort study from Sri Lanka indicated that liver levels of <4.5 g/dL Hb, >1300 µg/L serum ferritin, and >5 mg/g tissue ferritin were associated with poor survival; however, the median survival for Hb E/β-thalassaemia is 49 years. The study also showed that splenectomy did not eliminate the need for blood transfusion in 58% of cases [10]. As oxidative stress and iron overload are the main pathophysiological processes in thalassaemia, another study found that antioxidants (vitamin E group) and iron chelation increased haemoglobin by 10% at the end of 10 months and 11% at the end of 4 months, respectively, in non-transfusion-dependent patients [11]. A recent treatment strategy for Hb E/β-thalassaemia was based on the severity of the disease; for example, severe disease required lifelong blood transfusions and iron chelation to maintain haemoglobin at 9-10 g/dL, while milder forms required occasional transfusions to maintain the target haemoglobin levels [12].

## Conclusions

Peripheral blood smear in haemoglobin E/β-thalassaemia revealed target cells, teardrop-like cells, and basophil stippling, although they are not specific. Elevated Hb F by 29% was an ameliorating factor for disease severity. In a resource-limited setting, maintaining haemoglobin at 9.0 g/dL with shrinkage of splenic enlargement via folic acid and antioxidant vitamin supplements obviated the need for regular blood transfusions in this case.

## References

[1] Laman M, Aipit S, Bona C, Siba PM, Robinson LJ, Manning L, Davis TM (2015). Ultrasonographic assessment of splenic volume at presentation and after anti-malarial therapy in children with malarial anaemia. Malar J.

[2] Modell B, Darlison M (2008). Global epidemiology of haemoglobin disorders and derived service indicators. Bull World Health Organ.

[3] Olivieri NF, Pakbaz Z, Vichinsky E (2011). Hb E/beta-thalassaemia: a common & clinically diverse disorder. Indian J Med Res.

[4] Rund D, Rachmilewitz E (2005). Beta-thalassemia. N Engl J Med.

[5] Pani K, Sharma S, Murari M, Yadav M, Phadke S, Agrawal S (2018). Clinico-hematological profile of Hb E-beta thalassemia-prospective analysis in a tertiary care centre. J Assoc Physicians India.

[6] Sripichai O, Makarasara W, Munkongdee T (2008). A scoring system for the classification of beta-thalassemia/Hb E disease severity. Am J Hematol.

[7] Peters M, Heijboer H, Smiers F, Giordano PC (2012). Diagnosis and management of thalassaemia. BMJ.

[8] Allen A, Fisher C, Premawardhena A (2010). Adaptation to anemia in hemoglobin E-ß thalassemia. Blood.

[9] Rees DC, Porter JB, Clegg JB, Weatherall DJ (1999). Why are hemoglobin F levels increased in HbE/beta thalassemia?. Blood.

[10] Premawardhena AP, Ediriweera DS, Sabouhanian A (2022). Survival and complications in patients with haemoglobin E thalassaemia in Sri Lanka: a prospective, longitudinal cohort study. Lancet Glob Health.

[11] Yanpanitch OU, Hatairaktham S, Charoensakdi R (2015). Treatment of β-thalassemia/hemoglobin E with antioxidant cocktails results in decreased oxidative stress, increased hemoglobin concentration, and improvement of the hypercoagulable state. Oxid Med Cell Longev.

[12] (2022). Guidelines for the Management of Transfusion Dependent Thalassaemia (4th Edition - 2021). https://thalassaemia.org.cy/publications/tif-publications/guidelines-for-the-management-of-transfusion-dependent-thalassaemia-4th-edition-2021/.

